# Telomere length in subjects with and without SARS-CoV-2 infection: a systematic review and meta-analysis

**DOI:** 10.1590/1806-9282.20240387

**Published:** 2024-09-13

**Authors:** Faustino Ramón Pérez-López, Ana Maria Fernández-Alonso, Juan Ramiro Ulloque-Badaracco, Vicente Aleixandre Benites-Zapata, Seshadri Reddy Varikasuvu

**Affiliations:** 1 University of Zaragoza, Aragón Health Research Institute, Faculty of Medicine – Zaragoza, Spain.; 2 Torrecárdenas University Hospital, Departament of Obstetrics and Gynecology – Almería, Spain.; 3 Universidad Peruana de Ciencias Aplicadas, Faculty of Health Sciences – Lima, Peru.; 4 Universidad San Ignacio de Loyola, Research Unit for the Generation and Synthesis of Health Evidence, Vice-rector for Research – Lima, Peru.; 5 All India Institute of Medical Sciences, Department of Biochemistry – Deoghar, India.

## INTRODUCTION

Telomeres are nucleoprotein complexes containing repetitive DNA sequences that protect chromosomes from damage, senescence, metabolic disorders, stress, and cell death. They are considered markers of cell damage accumulation^1^. The nucleoprotein structures of telomeres at the chromosome ends maintain genome stability, determining the maximum of cell divisions. Telomeres possess properties that make them suitable as biomarkers in several diseases or conditions, including cardiovascular disease, cancer, stress, aging, and mortality. Telomerase is a reverse transcriptase enzyme that regulates telomere length (elongation and shortening), adding telomere repeat sequence to the end of telomeres^1,2^. The severe acute respiratory syndrome coronavirus 2 (SARS-CoV-2) pandemic has transformed quotidian and social life, medicine, research approach, and ­economy. The coronavirus disease 2019 (COVID-19) displays a wide range of heterogeneous clinical manifestations, from ­asymptomatic to acute respiratory syndrome or multi-organ failures, with lymphopenia, increased neutrophil count, and fibrinogen levels being common features of patients^3^.

Short telomere length is considered a risk factor for ­infections in general that can favor inflammation and age-­related ­immunity defects, long-term infections, and mortality. Telomere length has been reported to be shorter in individuals with complicated forms of SARS-CoV2 infection as compared with mild forms^4^. Age is among the strongest risk factors related to COVID-19 mortality, and lymphopenia is one of the common ­characteristics of the disease and is linked to the ­individual prognosis. Despite all those studies relating clinical severity with reduced telomere length, there is no clear information concerning the telomere length in subjects with and without SARS-CoV-2 infection. Therefore, this systematic review and meta-analysis address this scientific issue, and the protocol was registered at the PROSPERO-University of York (CRD 42023355542).

## METHODS

**Figure 1:**
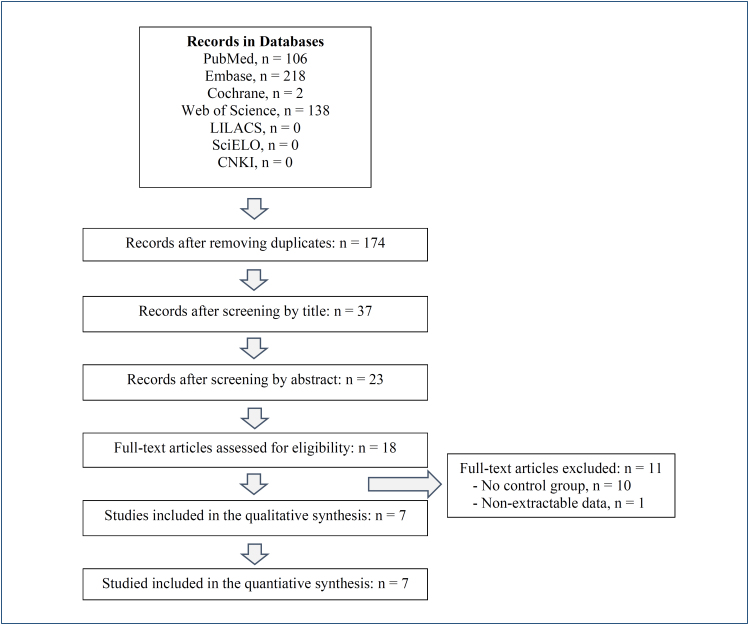
Identification of studies and flow diagram of the review process.

From 2019 to November 25, 2023, a comprehensive search was conducted in the following databases: PubMed, Embase, Cochrane, Web of Science, Literatura Latino Americana e do Caribe em Ciências da Saúde (LILACS), SciELO, and China National Knowledge Infrastructure (Figure 1). No limitations were placed during the search. The following keywords were used for this search: "telomere length," "telomerase," "telomerase activity," "COVID-19," "coronavirus disease 2019," "SARS-CoV-2," and "severe acute respiratory syndrome coronavirus."

We searched observational studies that evaluated the telomere length or telomerase activity in COVID-19 patients and uninfected subjects by SARS-CoV-2 (control group) between December 2019 and November 25, 2023, with validated laboratory tests or consistent clinical definition of the healthy control group. We excluded case reports, systematic reviews, scoping and narrative reviews, conference abstracts, and other publications lacking primary data. After the elimination of duplicates, the titles and abstracts were independently reviewed by three authors. Articles with all inclusion criteria were assessed for their content extraction (Figure 1). The retrieved literature was also reviewed for the identification of additional studies. The following information was obtained from the articles: title, author, country, year of publication, study design, age, sample size, methods of telomere length or telomerase measurement, and main clinical ­findings. The variables reported as ­medians and interquartile ranges were converted into mean and standard deviation. The mean was estimated by the formula x=(a+2m+b)/4 using the values of the median (*m*), percentile 25 and 75 (*a* and *b*, respectively).

The quality of the selected studies was assessed independently by two authors using the Newcastle-Ottawa Scale (NOS). This instrument consists of three parameters of ­quality: selection, comparability, and exposure/outcome assessment. Studies with scores ≥ 7 were considered as having a low risk of bias (high ­quality), scores of 5–6 as having a moderate risk of bias, and scores ≤4 as having a high risk of bias (low quality)5. Disagreements were solved by reaching a consensus among the research group.

Statistical analyses were performed using Review Manager 5.3 (The Cochrane Collaboration, Denmark). The DerSimonian and Laird random effect model and inverse variance were used as we anticipated heterogeneity among studies. Forest plots are reported as standardized mean difference (SMD) and 95% confidence interval (CI). The heterogeneity was evaluated using the I^2^ statistic. Values equal to or greater than 70% were considered a sign of severe heterogeneity for the I^2^ statistic. Funnel plots and Begg's test were planned for assessing publication bias if 10 or more studies were available. One-study leave-out sensitivity analysis was planned to test the robustness of overall results^5^. The authors respected the scientific quantitative and qualitative methods and ethics of included studies in the meta-analysis.

## RESULTS

After removing duplicates, 174 abstracts reported telomere length or telomerase activity information. After screening by titles, 37 items remained, and after reading 23 abstracts, there were 18 items for full content assessment (Figure 1). There were 10 full-text articles not including uninfected people studied with validated laboratory SARS-CoV-2 testing (control group), and one study reported non-extractable results. Finally, seven studies provided adequate information on telomere length in patients with SARS-CoV-2 infection and uninfected subjects^6-12^. There were no studies reporting telomerase activity in SARS-CoV-2 infected and uninfected subjects.

Telomere length was measured in peripheral blood of SARS-CoV-2 infected and non-infected subjects by southern blotting in blood mononuclear cells^6^, DNA methylation-based estimator of telomere length^7^, or relative average length in peripheral blood leukocytes using quantitative real-time PCR^8-12^. Two studies reported laboratory-tested patients with SARS-CoV-2 infection, while the control group included healthy subjects without SARS-CoV-2 ­laboratory testing^7,10^. The ­characteristics of the seven included studies, ages, and clinical information are summarized in Table 1. The NOS was used to assess the risk of bias, rendering five studies low risk^6,8-10,12^ and two studies moderate risk of bias^7,11^.

**Table 1:** Authors, study location, Newcastle-Ottawa Scale scores, SARS-CoV-2 infected and uninfected subjects, telomere measurement methods, and sensitivity analyses of study in which telomere length was measured and source of uncertainty by omitting one study at the time (I2).

Authors and reference	Location and NOS score	COVID-19 patients	Control group subjects	Telomere length measurement methods	Sensitivity analyses: Z scores (p-values); I2
Benetos et al.^6^	France. NOS: 7	17 severe COVID-19 (+) cases aged 87±8 years	21 non-COVID-19 aged 87 ± 9 years	Southern blotting	3.66 (0.0003); 93%
Cao et al.^7^	Spain and Germany. NOS: 5	407 COVID-19 patients	232 uninfected people.	Telomere length using 5 epigenetic clocks	6.24 (<0.00001); 67%
Santos et al.^8^	Brazil. NOS: 7	COVID-19 patients (n=41): Age range: 39.8 ±11.3 to 56.1 ±11.7 years	Uninfected subjects n=12	PCR in pharyngeal swab	3.02 (0.003); 91%
Krasnienkov et al.^9^	Ukraine. NOS: 7	64 patients of reproductive age recovering from COVID-19	42 uninfected subjects of the same age	PCR on venous leukocytes	3.08 (0.002); 92%
Mongelli et al.^10^	Italy. NOS: 8	117 post-COVID-19 survivors. Age: 67.2±10.9 years	144 non-infected subjects aged 62.5±9.0	PCR on leukocyte telomere length	3.03 (0.02); 92%
Retuerto et al.^11^	Spain. NOS: 5	251 hospitalized patients, median age 49 (range 7–96) years	169 healthy subjects with a similar age range	PCR on leukocyte telomere	2.96 (0.003); 91%
Savrun and Dirican^12^	Turkey. NOS: 4	70 patients aged 18 and over having positive SARS-CoV-2	70 negative PCR analysis aged 18 years and over	Telomere length by PCR	3.29 (0.001); 93%

**Figure 2:**
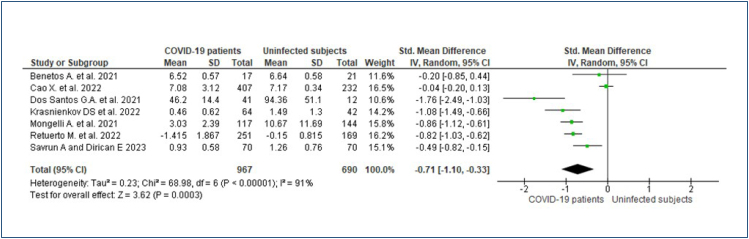
Forest plot comparing participants with and without SARS-CoV-2 infection (standardized mean difference) for telomere length, using the random-effect and DerSimonian and Laird methods.

The meta-analysis of a total of 1,604 participants (967 SARS-CoV-2 patients and 690 uninfected subjects) showed that SARS-CoV-2 infected patients have significantly shorter telomere length than non-infected subjects (SMD=-0.79, 95% confidence interval [CI]: -1.16, -0.17; p<0.001; Figure 2). There was high heterogeneity among studies (I^2^=91%), and a ­significant overall effect (Z=2.62, p=0.009). The sensitivity analysis demonstrated the same trend by deleting one study at a time, being the heterogeneity higher than 90%, and reduced to 67% by deleting the Cao et al.^6^ study (Table 1). Subgroup analyses or the funnel plot to study publication bias were not feasible due to few available studies. There are no publications concerning telomerase activity in subjects with and without SARS-CoV-2.

## DISCUSSION

This systematic review and meta-analysis demonstrated that SARS-CoV-2 infected patients displayed reduced telomere length compared with uninfected subjects. Our findings show a high degree of heterogeneity which is related to several ­factors, ­including the wide clinical spectrum and heterogeneous ­characteristics of the infection, age, comorbidity, immune response, and other factors that are constant in this particular viral infection. The statistical heterogeneity was high, although the effect was related to a particular study^7^, and when it was omitted, the heterogeneity was reduced.

Telomere length and telomerase activity seem to play a significant role in the human immune system, cellular vitality, inflammatory response, hypertension, diabetes, carcinogenesis, aging, and cell senescence^2,13,14^ that may favor the SARS-CoV-2 infection. Before the SARS-CoV-2 pandemic, telomere dynamics were related to chronic viral infection and immune exhaustion^15^, and short telomere length was associated with T-cell immunodeficiency and active DNA damage that may contribute to T-cell apoptosis^16^. Furthermore, during the SARS-CoV-2 pandemic, the severe course of this viral infection may accelerate the shortening of telomere as that related to age and epigenetic changes. Nalbant et al.^17^ compared blood count outcomes, infection biomarkers, and biochemical results in COVID-19-positive and -negative cases of people living in Brazil. They reported that the neutrophil/lymphocyte ratio has a role in the COVID-19 diagnosis, showing that there was no gender difference in the immune response between men and women with SARS-CoV-2 positivity. Soraya and Ulhaq^18^ meta-analyzed laboratory blood parameters in patients with SARS-CoV-2 pneumonia versus non-COVID-19 subjects, reporting lower leukocyte, neutrophil, and platelet counts comparing the infected versus the non-infected population, while lymphocyte, D-dimer, and C-reactive protein had no diagnostic value.

Severe COVID-19 survivors display telomere ­shortening and are older with comorbidity, more intense inflammatory response, and biological aging acceleration. However, the Haridoss et al.^19^ meta-analysis questions such association between telomere length and COVID-19 severity (severe versus mild cases). In our meta-analysis, the age and clinical manifestations of COVID-19 patients were very heterogeneous; some subjects had mild symptoms, while others experienced severe diseases and hospitalization risks. Age and immunodepression are major risk factors independent of age-related comorbid conditions. Telomerase activity is silent in many somatic cells and its activation can elongate telomeres, although its activation is insufficient to prevent telomere shortening^20^. Unfortunately, there were no telomerase activity results to be meta-analyzed in this study.

### Strength and limitations

This is the first global assessment of available evidence concerning telomere length in SARS-CoV-2 infected patients compared with uninfected subjects, showing shorter telomere length in infected cases. This meta-analysis has some limitations : The *first* is the heterogeneity of the studied population in terms of age, basal health, co-morbidity, clinical symptoms, and individual characteristics. *Second*, there was no information about telomerase activity and inflammatory markers in sufficient ­studies comparing infected and uninfected SARS-CoV2 subjects. *Finally*, we could not perform a meta-regression analysis due to the few available studies. Despite limitations, we demonstrated that SARS-CoV-2 infected patients had shorter telomere length than uninfected subjects. Future studies are needed to report telomere length, telomerase activity, inflammatory markers, and death risk considering symptoms, respiratory syndrome, and co-morbidity results in infected and uninfected subjects.

## CONCLUSION

Patients with SARS-CoV-2 infection displayed significantly shorter telomere length than uninfected subjects. Future research should determine the immune and inflammation response and telomerase activity in subjects with and without SARS-CoV-2 infection. Furthermore, inflammatory response during ­different phases of the infection needs to be appropriately and independently assessed including epigenetic modifications.

## ETHICAL APPROVAL

This study was a meta-analysis of published results and did not require ethical approval by a research ethics committee.

## Data Availability

The data that support this study are available from the meta-analyzed studies^6-12^ and this manuscript.
